# Influence of BPA exposure, measured in saliva, on childhood weight

**DOI:** 10.3389/fendo.2022.1040583

**Published:** 2022-12-08

**Authors:** Leticia Heras-González, Diana Espino, Maria Jose Jimenez-Casquet, Alejandro Lopez-Moro, Fatima Olea-Serrano, Miguel Mariscal-Arcas

**Affiliations:** ^1^ IBS.Granada, University Hospital of Virgen de las Nieves, Granada, Spain; ^2^ Department of Nutrition and Food Science, University of Granada, Granada, Spain

**Keywords:** BPA, saliva, childhood, weight, endocrine disruptors

## Abstract

**Introduction:**

Endocrine disruptors such as bisphenol A (BPA), BPA glycidyl methacrylate, and other BPA acrylate–based derivatives have been related to type 2 diabetes, the metabolic syndrome, and obesity, among other metabolic disorders. The objective of this study is to examine the influence of BPA exposure by saliva analysis and daily physical activity on the risk of overweight/obesity in schoolchildren from southern Spain.

**Methods:**

The study included 300 children (53.5% girls) aged 7–10 years. Participants completed a questionnaire with four sections: *participant data*, including demographic information and life and family habits; *semi-quantitative food frequency questionnaire*; *anthropometric variables*; and *physical activity variables.* All participants underwent dental examination, when the presence of sealants/composites in each tooth and other dental alterations was recorded, and samples of whole saliva were collected for UHPLC-MS/MS analyses.

**Results:**

Risk of overweight/obesity was significantly influenced by body fat composition (OR = 10.77), not walking to and from school (OR = 1.38), lesser energy expenditure in sedentary activities (OR = 12.71), greater energy expenditure in sports (OR =1.62), and exposure to BPA from dental sealants/composites (OR = 1.38; p = 0.058).

**Discussion:**

Further research is warranted on this issue in children, who may be especially vulnerable to the negative health effects of endocrine disruption.

## 1 Introduction

Many chemicals, both natural and artificial, can mimic with the endocrine system, and they are called endocrine disruptors (EDs); these chemicals are related to developmental, reproductive, brain, immune, and other problems (1). Some EDs can be accumulated for years in animal and human tissues. Others that degrade rapidly in the environment and in living beings may only be present for short time periods but can have severe effects if exposure happens during crucial periods of development (2, 3). The variety, robustness, and low cost of *polymer material* components have given them a key role in products related to public health, medicine, and dental composites and sealants (4, 5). EDs such as bisphenol A (BPA), BPA glycidyl methacrylate (bis-GMA), and other BPA acrylate–based derivatives have been related to endocrine disorders, including estrogen-like effect, type 2 diabetes, metabolic syndrome, and obesity, among other metabolic disorders (6–11). Estrogenic properties of BPA were reported in 1936 (12). Human exposure to BPA is universal, and unconjugated BPA molecules have been found in human biological samples (blood, tissues, urine, saliva, and milk) (9, 13–15).

Animal investigations and epidemiological studies have demonstrated that environmental exposure to EDs can favor adipogenesis and obesity (16–23).

Exposure to BPA and its analogs is of interest from the point of view of the use of composites and sealants that, due to migration from the polymer, appear in the saliva of treated dental patients. Our Research Group (24) carried out a study with healthy volunteers to whom dental sealant was applied, demonstrating a migration of BPA and derivatives to saliva, as well as its estrogenic effect through the E-Screen assay. Numerous works have subsequently confirmed again the migration from sealants and composites of BPA and derivatives; studies were carried out on adults and children (4, 25, 26). If BPA is determined in the saliva of subjects treated with composites/sealants, then these molecules are likely to pass into the digestive tract and, together with other sources of exposure to BPA, reach the urine, where it is frequently determined in adult subjects and children.

BPA can be part of dental sealants and composites in three ways: as a direct component, as a decomposition by-product of other ingredients [e.g., BPA glycidyl methacrylate (bis-GMA) and BPA dimethacrylate (bis-DMA)], or as a trace material from their manufacture (27). The most substantial window of potential dental exposure to BPA is directly after the application of resin-based dental sealants and composites (24–34).

A “temporary” BPA tolerable daily intake (TDI) of 4 µg/kg body weight (bw) a day has been established by European Food Safety Authority (EFSA) (2022) (35), concluding that people with medium and high exposure to BPA in all age groups exceed the new TDI. EFSA (35) has re-evaluated the risks of BPA in food and suggests significantly reducing the TDI compared to its previous assessment in 2015. In its draft of BPA, EFSA’s expert panel has established a TDI of 0.04 ng/kg of bw/day. The reduction of TDI results in the evaluation of studies has emerged in the literature from 2013 to 2018, particularly those indicating adverse effects of BPA on the immune system (36). Comparing the new TDI with estimates of consumer exposure to BPA in the diet, EFSA concludes that those with both average and high exposure to BPA in all age groups exceed the new TDI, indicating health concerns (35). Nutritional epidemiology studies have identified population groups at risk of pollutants exposure associated with non-transmissible chronic diseases (37, 38). The determinants contributing to obesity appear to be excessive energy intake (EI), insufficient physical activity (PA), sleep deprivation, and more stable temperatures in the home, among other features of modern lifestyles. The predisposition to obesity may be partly attributable to genetic factors although these cannot explain the current global obesity epidemic (39).

Organisation for Economic Co-operation and Development (OECD) projects a steady increase in obesity rates, which are expected to be particularly high in the USA, Mexico, and New Zealand (40%, 33%, and 32.2%, respectively, are expected to be obese in 2030). This increase is expected to be lower in countries such as Italy or Korea (13% and 9%, respectively, in 2030), whereas the predicted percentage in France and Spain is about 17% and 16.7%, respectively. Obesity accounts for 5%–10% of total health expenditure in OECD countries (OECD, 2017). Prevention is crucial to the problem of obesity in children by changing dietary and PA habits and minimizing exposure to environmental obesogens. Age group between 3 and 12 years is particularly critical, as they are more receptive to advice and adopting new habits (40, 41).

The aim of this study is to examine the influence of BPA exposure measured in saliva together with daily PA and EI on the obesity risk assessment in schoolchildren from southern Spain.

## 2 Material and method

### 2.1 Study population

The study included 300 schoolchildren (53.5% girls) aged between 7 and 10 years (inclusive) from educational centers in two provinces of southern Spain (Granada and Malaga). Written informed consent was obtained from parents/guardians of all participants in the study, which was approved by the Research Ethics Committee of the Andalusian Public Health Service.

### 2.2 Material

Participants completed an encoded questionnaire with four sections: *participant data*: sex, age, educational center, school year, and life and family habits, among others; *semi-quantitative food frequency questionnaire* (FFQ): recording the frequency (times/month, week, or day) and amount (weight/portions) of food consumption with reference to the previous 12 months, estimating energy from the data with the Dial program (Copyright ^©^2015 Alce Ingeniería) (42); *anthropometric variables*: weight, height, and waist circumference, classifying each participant as normal weight, overweight, or obese according to the BMI-based classification of Cole et al. (43); and *PA variables*: hours of sleep, method of going to school (walking, car, bicycle, etc.), hours/week of physical education in school, and extracurricular sports activities. Weight (in kilograms) was determined using a floor scale (model SECA 872; Hamburg, Germany) barefoot and wearing light clothes, and height (in meters) was measured with a stadiometer (model SECA 214 (20–207 cm) and waist circumference (in centimeters) with a measuring tape (model SECA 201), following procedures described in the CDC Anthropometry Procedures Manual (https://www.cdc.gov/nchs/data/pdf). Body fat percentage was calculated using the equations proposed by Marrodán et al. (44).

In the dental examination, one of the two professional dentists used a standard data collection form to record the presence of sealants or composites in each tooth and any other alteration (caries, lack of teeth, etc.), following the same procedure. The saliva sample was taken in the morning hours of the schoolchildren between the beginning of classes (9:00) until recess time (11:00) so that, during this period, they would not have eaten food or drinks that leave residues potentially contaminated by BPA.

Samples of whole saliva were collected by asking subjects to spit into pre-weighed glass flasks, adding 0.5 ml of methanol and freezing at −30°C (Biomedical Freezer Sanyo, model MDF-U333) until their analysis using a modification of a previously reported method (24).

#### 2.2.1 Material for BPA analysis

All chemical products and reagents were supplied by Sigma-Aldrich^®^ (Munich, Germany), including BPA (≥99%; Pm = 228.29 g/mol; CAS: 80-05-7) and Methanol Chromasolv^®^ for HPLC (≥99.9%).

### 2.3 Analytical method

UHPLC-MS/MS analyses were carried out by the Central Research Services of our university (45) using a Waters Acquity UPLCTMH Class system (Waters, Manchester, UK). Separation of compounds was performed on ACQUITY UPLC^®^BEH C18 column (1.7 µm; 2.1 × 50 mm) (Waters, Manchester, UK). A XevoTQ-xS tandem quadrupole mass spectrometer (MS) (Waters) equipped with StepWave ion guide and an orthogonal Z-spray UniSpray source was used for mass analyses. Statgraphics, Minitab, and Excel software were used for statistical treatment of data. MassLynx software 4.1 was used for UHPLC-MS/MS instrument control, peak detection, and integration. (BPA limits was Limit of Detection (LOD): 0.3 ng/g, Limits of Quantification (LOQ); 0.9 ng/g).

Chromatographic separation was performed using a binary gradient mobile phase consisting of 0.1% ammonia (solvent A) and MeOH (solvent B). Flow rate was 350 µl min^−1^, and the column was maintained at 40°C. Injection volume was 10 µl. Gradient program was as follows: initial, 30% B; 0–5.0 min, 10%–90% B; 5.1–7.0 min, 100% B; 7.05 min, 30% B. The MS was operated with electrospray ionization in negative ion mode. Instrument operating parameters were as follows: source temperature, 150°C; desolvation temperature 650°C; cone gas flow, 150 L h^−1^; desolvation gas flow, 500 L h^−1^; collision gas flow, 0.15 ml min^−1^; and nebulizer gas flow, 7.0 bar. BPA transition for quantification cone (V): 227.20 > 211.96 22. BPA transition for confirmation cone (V): 227.21 > 92.40.

#### 2.3.1 Saliva analysis

**Figure 1:**
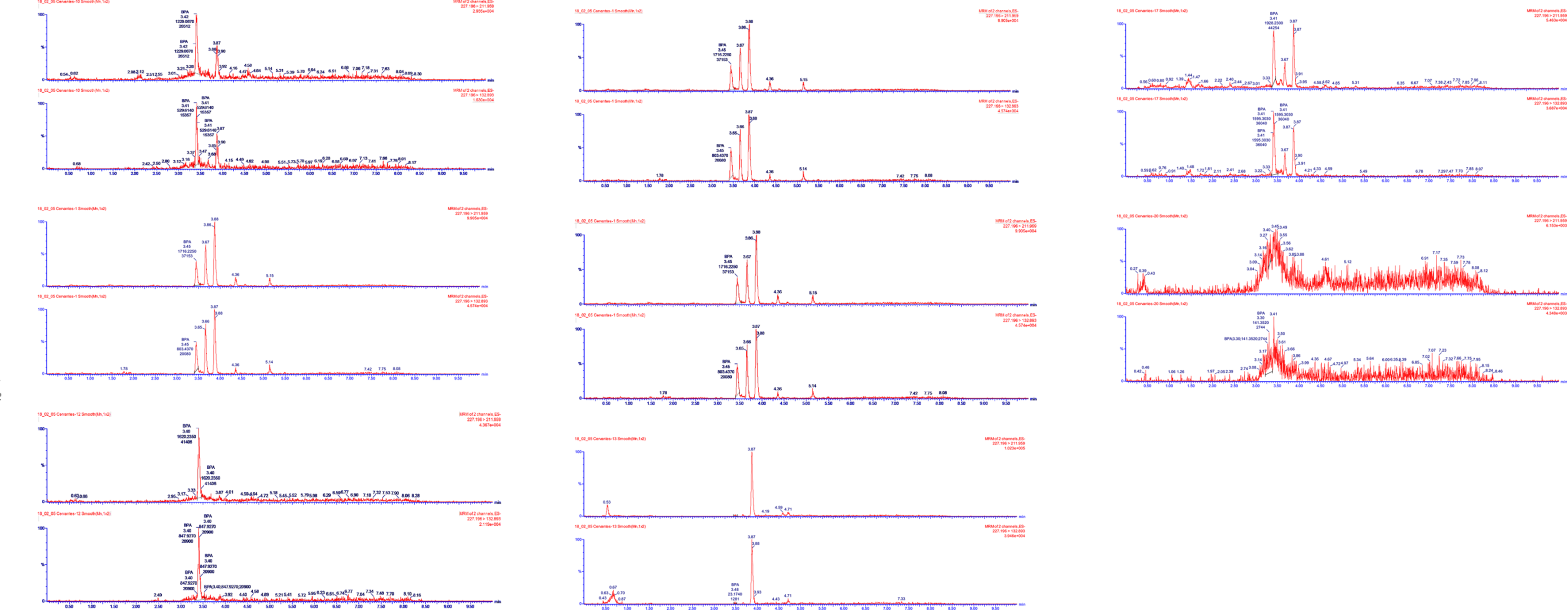
Chromatograms of the saliva samples and BPA reference standard. Saliva samples analyzed and confirmed by UHPLC-MS/MS.

After defrosting the flasks with saliva at room temperature, the amount of saliva was calculated by weighing the samples and subtracting the weight of the empty flask and of the 0.5 ml of methanol added. Saliva samples were then centrifuged (1,000g, 10 min) and passed through a 0.45-pm pore filter Millipore HDPE previously humidified with methanol. Aliquots of 10 µl were chromatographed. **Figure 1** depicts chromatograms of the saliva samples and the BPA reference standard.

### 2.4 Statistical analysis

SPSS version 22.0 (IBM, Chicago, IL) was used for the statistical analysis. After a descriptive analysis to calculate the means, standard deviations (SD), medians, and maximum and minimum values, logistic regression analysis was applied, as specified in the table footnotes. P < 0.05 was considered significant.

## 3 Results

**Table 1** summarizes the report made by dentists on dental alterations treated and untreated with composites/sealants.

**Table 1:** Number of damaged, treated, and untreated teeth of the 300 children participating in the study.

Caries frequency	% (N)
Children no teeth with caries	37.0 (110.0)
Children no treated teeth	43.0 (129.0)
Children treated teeth	20.4 (61.0)
Treatment frequency	
1–3 treated teeth	16.0 (48.0)
>3 treated teeth	4.7 (14.0)
1–3 teeth with caries	20.4 (61.0)
>3 teeth with caries	22.7 (68.0)

It is observed that the number of caries ranges from a single carious tooth per subject to a maximum of 10 damaged teeth, and without dental treatment, this represents 129 subjects. The treated teeth are a total of 61.0, corresponding between 1 and 3 carries to 16% of the population and 14% treated teeth corresponding to a value between 4 and 10 teeth per subject. Children without caries account for 110 subjects, which is 37% of the population studied.

**Table 2** lists the characteristics of the study population (aged between 7 and 10 years). The mean (SD) Body Mass Index (BMI) was 18.50 kg/m^2^ (SD: 2.94); 10.5% of the children were obese. On the basis of the daily activity questionnaire, the mean energy expenditure (TEE) was 1,215.01 kcal/day (SD: 316.75), according to the formula children proposed by Harrell et al. and Ridley et al. (46, 48). The mean EI (kcal/day) calculated from the FFQ was 1,282.73 kcal/day (SD: 392.95). Concentration of BPA in saliva was between not detected (ND; 75.32 ng/ml) and mean (29.59 ng/ml; SD: 17.07) (**Table 2**).

**Table 2:** Population characteristics (n = 300).

	Minimum	Maximum	Mean	SD
Age (years)	7.00	10.00	8.92	1.01
Weight (kg)	17.85	66.40	34.40	8.49
Height (m)	1.16	1.69	1.35	0.081
BMI (kg/m^2^)	14.24	29.16	18.50	2.94
Waist circumference (cm)	48.00	94.00	63.65	7.98
Waist-to-height ratio	0.37	0.64	0.47	0.046
Fat composition* (Fat %)	11.11	35.50	23.25	5.00
Hours sleeping	7.50	14.00	8.46	1.08
MET sleeping (kcal/weight/night)	166.40	679.80	295.14	79.38
Hours walking to school	0.00	0.50 (30´)	0.24 (15´)	0.25
MET kcal journey to school/weight	25.88	96.28	51.70	13.33
PA hours of physical education	0.00	7.00	1.88	0.55
MET PA (kcal/weight)	0.00	988.61	192.03	78.39
Moderate activity up to 24 h	6.21	16.00	13.10	1.35
MET sedentary/day/weight**	262.40	1290.10	643.81	180.13
Total 24-H MET (kcal/day)	588.16	2673.65	1215.01	316.75
Intake (kcal/day)	602.50	3016.40	1282.73	392.95
No. composite/sealant treatments	0.00	10.00	1.68	2.12
Nanogram of BPA/ml of saliva	ND***	10.76 to 75.32	29.59	17.07
% Participants in each BMI category	Normal weight	Over weight	Obesity	
	62.70	26.80	10.50	p < 0.001

*Fat % calculated as 106.50 × WHI − 28.36 for boys and 89.73 × WHI − 15.40 for girls (44). ** MET data for different degrees of activity were based on the values proposed by Harrell et al. (46) and Ridley et al. (47) *** ND, not detected.

After analyzing the study population characteristics that may be related to the weight status, a statistically significant OR for normal weight was obtained for energy expenditure in various activities, e.g., walking to school, extracurricular PA, and EI in daily diet, whereas a trend toward an increased likelihood of obesity was observed for BPA exposure overall and for BPA exposure from oral composites and sealants (exposure vs. non-exposure) in particular, as shown in **Table 3**. The logistic regression adjustment to determine the goodness-of-fit of the model according to the chi-squared test (p < 0.001) explains 85.1% of the effect of the independent variables on the dependent variable.

**Table 3:** Logistic regression analysis to determine factors associated with normal weight/overweight.

Normal weight/overweightReference category is overweight	B	p	OR	95% confidence interval for Exp(B)	
				Lower limit	Upper limit
Male	−0.229	0.431	1.008	0.733	1.387
Ref. female					
% Body fat	2.933	0.001	10.770	5.89	19.70
Ref > median value					
Walking to school < 30 min/day	0.324	0.047	1.382	1.005	1.902
Ref > 30 min/day					
Daily sports activity < MET (46.2 MET)	−0.218	0.003	1.614	1.171	2.223
Ref, > MET					
< sedentary MET/day	0.619	0.001	12.713	8.487	19.041
Ref > sedentary MET/day					
MET_24H_ < median (1154.27)	2.289	0.001	8.911	4.738	16.758
Ref.MET_24H > median					
Estimated kcal intake/day <median (1067 kcal/day)	0.529	0.075	1.696	0.948	3.035
Ref > median					
No detected in saliva BPA	−0.393	0.058	1.381	0.938	1.763
Ref: detected					

## 4 Discussion

Overweight/obesity is currently considered a non-communicable disease (NCD) and dependent on multiple factors (37, 38). In this work, some of these factors have been considered, such as EI, PA, and exposure to an ED classified among other effects as obesogenic, and a direct source of exposure to it has been identified, by measuring BPA in saliva and by checking the existence of composites/sealant in the mouth of the studied subjects.

The degree of obesity in this study population is estimated at 10.5%, consistent with the OECD values for the Spanish school population (40). A factor considered to partially explain the obesity situation of schoolchildren in southern Spain has been the average intake of sugar (49); in addition, factors such as EI and PA have been considered. Exposure to ED, considered estrogenic and obesogenic, allowed estimating risks of obesity in schoolchildren between 7 and 10 years old, estimating the relationship between obesity and exposure to phytoestrogens and BPA through diet (15).

Dental situation of the study population is a determining factor in estimating oral exposure to BPA. This group of 300 schoolchildren shows that 20.4% of teeth treated with composites/sealants and 43% of subjects have dental caries that can be treated with composites, whereas 37% of subjects do not have caries. A previous review carried out a few years earlier in the same region and only with 7-year-old children showed values of 46.9% of subjects with caries, the situation has not changed significantly, although it was estimated that 60.0% of the population had received some dental care (50).

This study considers lifestyle variables and BPA exposure due to the migration of this monomer from polycarbonates and epoxy resins used in dental restoration materials depending on the number of teeth that were treated with sealant/resin composite (24, 26–31, 34). The 300 pre-adolescent children (aged 7 to 10 years) in this study had a mean of 29.59 ng of BPA/ml (SD: 17.07) of saliva, as confirmed by UHPLC-MS/MS, and 20.4% experienced direct oral contact with BPA from sealants or fillings exposure to BPA through the use of composites, and sealants has been revealed by identifying the subjects with dental treatment, analyzing the saliva of all the subjects under study in this work. The treated subjects account for n = 61 and the untreated n = 129, which present lesions susceptible to the use of composites or sealants and healthy subjects at the time of their dental checkup, which account for n = 110. In the current study, the exposure or not of BPA in the saliva of the population has been considered as a primary factor. In this case, a value of OR = 1.381 (p = 0.058) has been obtained. A significantly greater likelihood of an overweight condition was associated with the presence of BPA in saliva. Factors that emerged in this study as significantly contributing to explain overweight/obesity were body fat composition (OR = 10.77), not walking to and from school (OR = 1.382), lesser energy expenditure in sedentary activities (OR = 12.713), and greater energy expenditure on sports activities (OR =1.614).

This study considered the life and nutritional habits of schoolchildren and of BPA exposure, in their saliva on a given day. Although these BPA values do not precisely represent the exposure of each individual, our findings demonstrate that greater exposure is related to a higher risk of overweight/obesity. The presence or absence of BPA and its influence on exposure to ED is of greater importance than its concentration in a saliva sample taken at a single time point. Although saliva is a biological sample that shows BPA exposure, this biological sample will not always be the only or the most appropriate sample to test for human exposure to BPA. Further research is warranted on this issue, and a study is currently under way by our group on BPA concentrations in the urine of this same population to gather more precise data on their exposure. In conclusion, it appears important to identify and minimize sources of BPA exposure, especially in populations whose age and lifestyles make them particularly vulnerable to undesirable responses to endocrine disruption. These results are limited to school children aged 7–10 years and do not take into account the nutritional and life habits that can be acquired in adolescence (49). Obesity and overweight are a consequence of numerous factors (48), and this study considers those relating to activity/sedentary patterns, daily EI, and exposure to a hormone disruptor (BPA).

## Data availability statement

The raw data supporting the conclusions of this article will be made available by the authors, without undue reservation.

## Ethics statement

The studies involving human participants were reviewed and approved by University of Granada. Written informed consent to participate in this study was provided by the participants’ legal guardian/next of kin.

## Author contributions

The study was designed by FO-S and MM-A; data were collected and analyzed by LH-G, MM-A, DE, MJ-C, and AL-M; data interpretation and manuscript preparation were undertaken by FO-S, LH-G, and MM-A. All authors contributed to the article and approved the submitted version.
